# Computational modeling of the negative priming effect based on inhibition patterns and working memory

**DOI:** 10.3389/fncom.2013.00166

**Published:** 2013-11-19

**Authors:** Dongil Chung, Amir Raz, Jaewon Lee, Jaeseung Jeong

**Affiliations:** ^1^Department of Bio and Brain Engineering, Korea Advanced Institute of Science and TechnologyDaejeon, Republic of Korea; ^2^Department of Psychiatry, Jewish General Hospital, Montreal Neurological Institute and McGill UniversityMontreal, QC, Canada; ^3^Department of Psychiatry, College of Physicians and Surgeons, Columbia UniversityNew York, NY, USA

**Keywords:** negative priming, disinhibition, Stroop task, working memory, parallel distributed processing

## Abstract

Negative priming (NP), slowing down of the response for target stimuli that have been previously exposed, but ignored, has been reported in multiple psychological paradigms including the Stroop task. Although NP likely results from the interplay of selective attention, episodic memory retrieval, working memory, and inhibition mechanisms, a comprehensive theoretical account of NP is currently unavailable. This lacuna may result from the complexity of stimuli combinations in NP. Thus, we aimed to investigate the presence of different degrees of the NP effect according to prime-probe combinations within a classic Stroop task. We recorded reaction times (RTs) from 66 healthy participants during Stroop task performance and examined three different NP subtypes, defined according to the type of the Stroop probe in prime-probe pairs. Our findings show significant RT differences among NP subtypes that are putatively due to the presence of differential disinhibition, i.e., release from inhibition. Among the several potential origins for differential subtypes of NP, we investigated the involvement of selective attention and/or working memory using a parallel distributed processing (PDP) model (employing selective attention only) and a modified PDP model with working memory (PDP-WM, employing both selective attention and working memory). Our findings demonstrate that, unlike the conventional PDP model, the PDP-WM successfully simulates different levels of NP effects that closely follow the behavioral data. This outcome suggests that working memory engages in the re-accumulation of the evidence for target response and induces differential NP effects. Our computational model complements earlier efforts and may pave the road to further insights into an integrated theoretical account of complex NP effects.

## Introduction

Negative priming (NP) refers to slowing down of the response for target stimuli that have been previously exposed, but ignored (Neill, 1977; Tipper, 1985; Tipper and Cranston, 1985). Frequently observed in selective attention tasks such as the Stroop task (Stroop, 1935), NP supports the dual-process view whereby relevant information is highlighted and irrelevant information is actively blocked [identity NP: (Schrobsdorff et al., 2012b), location NP: (Milliken et al., 1994; Park and Kanwisher, 1994), for reviews see (Fox, 1995; May et al., 1995; Mayr and Buchner, 2007)]. The possible neuropsychological mechanisms underlying NP include cognitive inhibition and memory retrieval. While the former assumes that inhibition of previously ignored information necessitates additional time for the “disinhibition”—release from inhibition—of the ignored information for subsequent processing (Tipper, 1985; Fuentes and Tudela, 1992; Engle et al., 1995; Malley and Strayer, 1995; May et al., 1995; Strayer and Grison, 1999; Grison and Strayer, 2001), the latter emphasizes the idea that the ignored information receives a “do not respond” tag, which causes a slowing of the subsequent response compared with the “respond” tag (Tipper et al., 1991; Neill and Valdes, 1992; DeSchepper and Treisman, 1996; Groh-Bordin and Frings, 2009; von Hecker and Conway, 2009). Other accounts for NP include selective attention (Moore, 1994b; Milliken et al., 1998), working memory capacity (Conway et al., 1999; Long and Prat, 2002), inhibition of return (Pratt et al., 1999; MacLeod et al., 2003), and feature mismatch (Lowe, 1979; Park and Kanwisher, 1994; MacDonald and Joordens, 2000). These effects emphasize the importance of biased attention and the automatic retrieval of information processing units in the preceding trial (prime trial). NP has been used to elucidate attention and memory functions and, particularly, to elucidate the interplay between them (MacLeod and MacDonald, 2000). NP is a valuable index for investigating attention and memory disturbances in normal aging (Titz et al., 2003) as well as neuropsychiatric disorders including, schizophrenia (Beech et al., 1989, 1991; Laplante et al., 1992; Williams, 1995, 1996; Salo et al., 1996; Macqueen et al., 2002), autism (Brian et al., 2003), and obsessive-compulsive disorder (OCD) (Enright and Beech, 1990, 1993a,b; Stein and Ludik, 2000).

Over the past two decades, theoretical accounts of NP have flourished e.g., the distractor-inhibition model (Houghton and Tipper, 1994), the episodic memory retrieval model (Neill and Valdes, 1992; Frings et al., 2007), temporal discrimination model (Milliken et al., 1998), the dual-mechanism hypothesis (May et al., 1995), and the computational imago-semantic action model (CISAM) (Schrobsdorff et al., 2007), but these theories remain controversial (Kane et al., 1997; Tipper, 2001; Grison et al., 2005a,b). This lack of consensus results not only from individual differences depend on demographics [age (Gamboz et al., 2002), sex (Bermeitinger et al., 2008), and neuropsychiatric disorders (Ungar et al., 2010)], but also from the complexity of the stimuli combinations in NP; the NP effect depends on subtle experimental parameters such as inter-stimulus time intervals, and various stimulus combinations within NP may represent different degrees of inhibition. Moreover, the dearth of predictive computational formulations is a substantial reason for this lacuna (Schrobsdorff et al., 2007). Thus, an integrated theoretical explanation that accounts for the effects of such parameters has been elusive [c.f., see Schrobsdorff et al. (2012a) for the general model for Negative priming (GMNP model)].

Therefore, the primary aim of this study was to investigate whether different degrees of NP effects are present during Stroop task performance depending on different combinations of prime-probe stimuli. The Stroop task, a benchmark experimental paradigm for selective attention, affords a good experimental platform for robust NP effects (MacLeod, 1992; Egner and Hirsch, 2005). In a classic Stroop paradigm, participants attempt to identify the ink color of word stimulus (Raz and Campbell, 2011). The stimuli are grouped into three Stroop conditions; words can be congruent (i.e., the ink color is congruent with the semantic meaning, as in the word “RED” printed in red ink), neutral (i.e., the ink color is largely immaterial to the semantic meaning, as in the word “LOT” printed in red), or incongruent (i.e., the ink color is incongruent with the semantic meaning, as in the word “BLUE” printed in red). Congruent items typically result in shorter reaction time (RT) than those elicited for neutral items (i.e., Stroop facilitation), and incongruent items usually result in longer RTs than those induced by neutral items (Stroop interference) (Stroop, 1935). Independent of the Stroop condition, NP occurs when the non-target (ignored) information of the previously given stimulus, called “the prime,” is repeated as the target information to respond at the currently given stimulus, “the probe,” during the task. Although the level of inhibition (or suppression in responses) to ignored items are equal, different pairs of successive stimuli (i.e., prime-probe pairs) among congruent, neutral, and incongruent items in NP trials may exhibit different speeds of recovery, disinhibition, in the ability to accumulate enough evidence to respond correctly to the target. We assumed that RTs would index both task performance as a function of Stroop conditions and degrees (or speeds) of disinhibition among NP subtypes categorized by Stroop condition of probe stimulus. To test the hypothesis, we defined three NP subtypes—NP with an incongruent probe (NP-I), NP with a neutral probe (NP-N), and NP with a congruent probe (NP-C)—and three non-NP subtypes, each corresponds to each NP subtype—contra-NP-I, contra-NP-N, and contra-NP-C. We measured RTs from each subtype and examined whether RTs for NP and contra-NP subtypes are statistically different. If each pair, e.g., NP-I vs. contra-NP-I, exhibit different RTs, it is concluded that NP exists for corresponding NP subtype. In the present study, we also showed significant RT differences among NP subtypes, suggesting the presence of differential disinhibition of the NP effect.

What is the origin of the differential disinhibition of the NP effect? Among the several potential origins of NP subtypes, we used computational models to examine whether differential disinhibition of NP arises from selective attention and/or working memory involvement during Stroop task performance (Conway et al., 1999, 2003; de Fockert et al., 2010). We constructed a formal parallel distributed processing (PDP) network model inspired by one of the earliest computational formulations of the Stroop effect (Cohen et al., 1990). The previously published formulation of the PDP model comprises a multilayer network that processes the information via activation spreading between units along pathways of different strengths. Presenting a stimulus activates input units corresponding to the word and color of the stimulus. Task demand units determine the degree to which “word reading” vs. “color naming” dominates subsequent processing. Eventually, a response is produced when one of the output units exceeds its threshold. In this model, interference occurs when two parallel, simultaneously activated pathways generate conflicting activation at their intersection, while facilitation arises when two pathways produce coinciding activation. Attention is realized as the modulation of the operation of processing units along a pathway. Thus, selective attention is intrinsic to the PDP model (MacLeod and MacDonald, 2000). However, this classic model does not account for the underlying mechanism of working memory regarding a stimulus from the previous trial, a mechanism that putatively involves the prefrontal cortex (PFC) (Fletcher et al., 1998; O'Reilly et al., 1999; Lepage et al., 2000) and that might assist in differential disinhibition (PDP does implement a long-term memory that stores the history of all previous sequences. The major difference in this aspect between PDP and our model will be discussed in the Discussion section). To account for this retrieval, we constructed a PDP model with working memory (PDP-WM), in a form of time delay neural network (TDNN) model (The source code of the model is available at the project website: http://raphe.kaist.ac.kr/NegativePriming/). We introduced additional temporal-storage units that interact with the output units. We compared the simulation results of differential NP disinhibition during Stroop task performance between the conventional PDP and our PDP-WM model.

## Materials and methods

### Ethics statement

All procedures in the current study were approved by the institutional review board of the Weill Medical College of Cornell University, New York City. After receiving an explanation of the procedures, participants provided written informed consent. The participants were compensated for their participation.

### Participants

Sixty-six (24 females) right-handed individuals who were proficient readers of English and aged 20–35 years (mean = 27 ± 3.6) were recruited through advertisements in New York City. All behavioral data were acquired from participants who were medical students in the Manhattan area, and some of the participants had participated in additional studies.

### Materials and apparatus

We used the materials reported by Raz et al. (2002) (for further information, see procedures therein). The participants sat at a viewing distance of ~65 cm in front of a color computer monitor. Stimuli sets consisted of a single word written in one of four ink colors (red, green, blue, or yellow) appearing at the center of the computer screen where a black fixation cross was visible. All characters were displayed in upper case font against a white background, and the stimuli subtended visual angles of 0.5° vertically and 1.3–1.9° horizontally (depending on word length). Two classes of words were used: color words (RED, GREEN, BLUE, and YELLOW) and neutral words (LOT, KNIFE, SHIP, and FLOWER); the latter class was frequency-matched as well as length-matched to the color words.

The task stimuli had three different conditions: a congruent condition consisting of a color word inked in its own color (e.g., the color word RED inked in red); a neutral condition consisting of an indifferent word inked in any one of the four colors (the word LOT inked in red); and an incongruent condition consisting of a color word inked in any of the three colors other than the one to which it referred (e.g., the color word BLUE inked in red). During each trial, the participants were asked to indicate the ink color in which a word was written by depressing one of four keys on a keyboard. The color-labeled response keys were V, B, N, and M for the colors red, blue, green and yellow, respectively. Two fingers of each hand were used to press these response keys (e.g., left middle finger for V and right index finger for N). Speed and accuracy were emphasized equally.

### Task design and procedure

An experimenter gave an instruction and stayed in the room during the task. The participants were asked to play a computer game (i.e., the Stroop task). They were instructed to respond as quickly and as accurately to the ink color of the visual stimuli. In all cases, the participants were instructed to focus on the center fixation cross and respond by depressing a key indicating the ink color of the stimulus.

The participants were instructed to focus their eyes upon a fixation cross at the center of the screen. A stimulus then appeared on the screen replacing the crosshair. The stimulus remained until the participants responded. Upon a response, veridical visual feedback was provided (i.e., CORRECT or INCORRECT was centrally flashed in black ink), and the fixation cross was redisplayed at the center for a variable duration contingent upon the subject's RT. If the subjects did not respond within 2 s, the screen also changed to the feedback screen. After the fixation screen, a new stimulus appeared on the screen, again replacing the crosshair and beginning the next trial. The inter-stimulus interval (ISI) was fixed to 4 s.

A full practice session (i.e., 144 trials) preceded the first measurement for each subject. This training session was used to confirm the participants' understanding on the instruction, response keys corresponding to each color, and to check their performance capability (RT and accuracy). Following this training session, the participants took a short break and then completed 144 experimental trials. One-third of all trials were congruent, neutral, and incongruent. Trial order was randomized throughout the experiment.

### Neural network models

**Figure 1:**
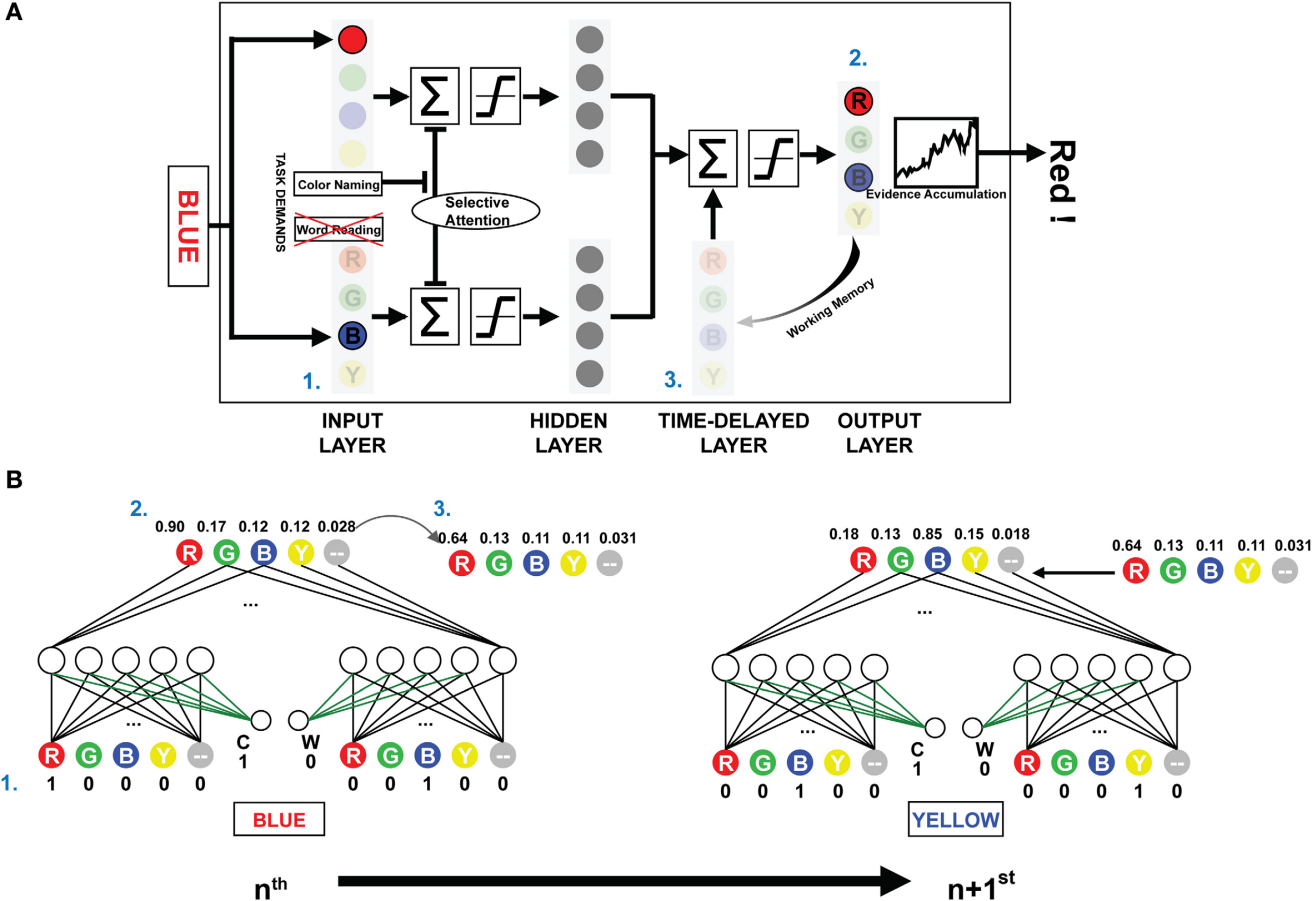
**The architecture and schematic diagram of PDP-WM. (A)** (1) The input layer encodes the provided stimulus, and the summated activations are transmitted to the next layers according to a non-linear function. The task demands compensated for the negative bias to represent selective attention. (2) Reaction time for the stimulus is calculated by the number iterations required for the activation of the evidence accumulator to exceed the response threshold based on the output layer activations. (3) The unit activities are stored in the time-delayed layer that affects processing of the subsequent stimulus. Nodes corresponding to the neutral words in all layers were omitted to simplify presentation. **(B)** The artificial neural networks depict the activities from processing two consecutive example trials for the NP effect (NP-I). Numbers next to each node show the unit activities. The green-colored weights transmit the task demand activity. Arrow-lined weights between the time-delayed layer and output layer determine the effect of working memory.

The present investigation used the PDP and PDP-WM computational models. The PDP model consisted of three layers: an input layer, a hidden layer, and an output layer. The PDP-WM had an additional time-delayed layer before the output layer (Figure 1A). Two task-demand units—one each for word-reading and color-naming tasks—characterized both the PDP and PDP-WM models. The input layer had four color units, each receiving color information (red, green, blue, and yellow, respectively), four word units, each receiving word information (RED, GREEN, BLUE, and YELLOW, respectively), and two non-color units that received the neutral words that were not related to color information (one each for color and word task to match the number of units even). The hidden layer had the same number of units as the input layer (10 units), and the output layer had five units for the responses RED, GREEN, BLUE, YELLOW, and neutral word (corresponding to all four neutral words). Presentation of a stimulus to the input layer activated each pertinent unit. The hidden (intermediate) layer received the activation information and transmitted it to the output layer. In the process of calculating the net input for the output layer, default inhibitory bias was provided to reproduce proper selective attention effects (−6 in the current study). This biased inhibition worked only in the hidden layer of ignored task. In the current study, the weights between task demand units and the units in the intermediate layer were fixed at 6. For example, during the color naming task, the task demand unit for the color naming was activated (set to 1), and the word-reading unit was deactivated (set to 0). Thus, in the selectively attended task, the activation from the task demand compensated the biased inhibition.

While the PDP model used running averages to generate the mean activity of all previous outcomes to affect the hidden and the output layer, the PDP-WM model contained the time-delayed layer that looked back over time to capture the temporal evolution of output patterns instead of using a running average. In the running average algorithm, the delay factor “1-τ” determined the degree of averaged net input for calculating the next net input using the formula:

(1) $$\bar{{\text{net}}_{j}}(t)=\tau {\text{net}}_{j}(t)+(1-\tau )\;\bar{{\text{net}}_{j}}(t-1),$$

where τ was a rate constant. In contrast, the PDP-WM had the time-delayed layer, which stored the previous activity of the output layer and thus provided feedback interactions to the net output of successive trials. Each “neuron” in the temporal-storage layer of the PDP-WM model was provided with a memory to store the previous outcome of the output layer (updated each trial). The look-back period of the PDP-WM caused the output layer to interact with the memory of previously developed patterns and produced the new output. The capacity of working memory in the PDP-WM was determined by weighted connections of the time-delayed layer and the output layer (*w*_delay_; see time-delayed layer section below). A large weight increased the effect of working memory on the response time of the succeeding stimulus. In the current study, the weight connecting the two layers was set to 0.8, indicating that 80% of the obtained outcomes were sustained through working memory.

The PDP-WM model was different from the conventional PDP model in two aspects: the running average algorithm was excluded, and the time-delayed layer was introduced instead. We constructed a PDP-refined model by discarding running average from the conventional PDP model. Hence, the PDP-refined model was identical to the PDP-WM except for the time-delayed layer. We used the PDP-refined model for two purposes. First, we used the model for training sessions under the assumption that the strength of working memory does not change during the Stroop task. Second, we compared the NP performances of the PDP-WM model with the PDP-refined model to examine whether working memory has critical role.

### Model equations

The current study used a backpropagation mechanism to train the model; backpropagation is one of the most frequently used learning algorithms. Our model used standardized algorithms (e.g., a logistic function for activity and an evidence accumulator) with default parameters except for additionally mentioned parameters).

#### Node activation with non-linear function

The activation of nodes included in the input layer was decided by the stimulus information. Each input stimulus had three pieces of information: color and word of the stimulus, and the task that participants were instructed to do (color naming). The activity of the nodes in the hidden and output layers was decided by the following non-linear equation:

$${\text{net}}_{j}(t)=\Sigma_{i}a_{i}(t)w_{ij}$$

(2) $$a_{j}(t)=\text{logistic}[{\text{net}}_{j}(t)]=\frac{1}{1+e^{s\;{\text{net}}_{j}(t)}},$$

where *a*_*j*_(*t*) is the activity, net_*j*_(*t*) is the calculated net value of *j*th node at time t, and *w*_*ij*_ is weight connecting *i*th node and *j*th node. Based on the previous studies of the PDP model (Cohen et al., 1990; Cohen and Servan-Schreiber, 1992), we set s, slope of the logistic function, as a constant (−1).

#### Evidence accumulator

As a response mechanism, we used an evidence accumulator that has also been used in PDP (Cohen et al., 1990). A response to the given stimulus is made when the accumulated activation exceeds the threshold (6.0 in the current study). For each trial, the accumulator adds a small random amount of total activated value; this random amount came from a normal distribution with a mean of μ and fixed standard deviation (*SD*) of σ, which was 0.1 in the current study. Here, the mean value was decided by the following equation:

(3) $$\mu_{k}=\alpha ({\text{act}}_{k}-\max\_{\text{act}}_{l\;\neq \;k}),$$

where α is the rate of evidence accumulation, 0.1, and act_*k*_ is the activation value of the *k*th output node. Thus, the accumulator adds predetermined proportion of the activation difference between the *k*th node and the maximum activation among the other output nodes, max_act_*l* ≠ *k*_. The number of iterations of the accumulator necessary to exceed the threshold corresponded to the RT in the human experiments (Figure 1A).

#### Time-delayed layer

There were five temporal-storage nodes corresponding to each color and one neutral word node. Outputs of the previous stimuli (at time *t*-1) were saved in the matched node (Figure 1B). The stored outputs were linearly added to net output of the successive object:

(4) $$\bar{{\text{net}}_{j}}(t)={\text{net}}_{j}(t)+\{\bar{{\text{net}}_{j}}(t-1)\times w_{\text{delay}}\},$$

where net_*j*_(*t*) is summation of corresponding hidden-layer nodes at time *t* and *w*_delay_ is a weight for each temporal-storage node. The working memory capacity was exhibited by changing the percentage of temporal storage. The time-delayed layer maintained the information regarding the prime stimulus, and net outputs at the time point of the response were stored (e.g., “RED,” a distractor from the prime, had the lowest activity, and “BLUE,” the target of the prime, had the highest activity; Figure 1B). As a result, the stored information regarding the prime was linearly projected to the output layer, which affected the subsequent decision making. Thus, the distractor from the prime stimulus suppressed the target from the probe, and it took more iterations (longer RT) for the model to make a response (i.e., for the evidence accumulator to exceed the threshold). The time-delayed layer consisted of 80% of the previous net output node as a default. The model should have sufficient working memory capacity, at least 40% in our model, to simulate the significant NP effect, which fits to the previous experimental research (Conway et al., 1999). All the source codes for computational models and simulations are available at the project website (http://raphe.kaist.ac.kr/NegativePriming/).

### Training and testing

All weights connecting the layers, except those for task-demand (*w*_*td*_) and those between the output and the time-delayed layer (*w*_delay_), were randomly initialized within the range of −0.5 to 0.5. Each weight gradually changed through a backpropagation learning algorithm (Rumelhart et al., 1986) with a fixed learning rate (0.03 in the current study) for word-reading and color-naming tasks. *w*_*td*_ and *w*_delay_ had constant values of 6 and 0.8, respectively. The ratio between word-reading and color-naming tasks used for the training and the task-demand weights (*w*_*td*_) affected the degree of selective attention and were determined empirically (4:1). We used a randomly generated set of stimuli based on the word/color ratio that had inputs for either word or color (e.g., “red, color-naming, NULL” or “NULL, word-reading, BLUE”). For neutral word learning, we provided four times as many items to match the human experiment condition: four different kinds of neutral words that corresponded to each color word in terms of length were used. Gaussian-distributed noise with an adequate *SD* was introduced to all units, except for the units in the input layer. A total of 100,000 randomly chosen stimuli were used for training, which resulted in a mean square error (average of the squared errors between outcome and target values in the output layer) of about 0.003. During training of the models, the input data for the word-reading task were four times larger than those of the color-naming task to implement the pre-experiences of the participants (Cohen et al., 1990). In first-language acquisition, learning simple words such as colors is not only restricted to a specific sequence, but occurs throughout long time period within various context, rules, and phonological cues (MacWhinney et al., 1989). To implement this condition, we implemented a PDP-refined model in the training session, omitting working memory effect during the first acquisition period. The time-delayed layer was attached after the training session. By introducing the working memory to the model (i.e., connecting the time-delayed layer), we can simulate the performance of the Stroop task participants. We used 100 epochs of test sets in which each of the epoch contained 1000 items of Stroop stimuli. The model was re-initialized and trained for each epoch of tests to show the robustness of our model to the differences between individual participants. The performance was measured by iteration of the model that was simulated by the evidence accumulator to respond for each given stimulus.

### Statistical analysis

Comparing the RTs in each subtype, incorrect responses were excluded from the analysis, and RTs three *SD*s above and below the mean of each Stroop condition were excluded as outliers. We used a One-Way analysis of variance (ANOVA) to assess Stroop and NP effects (RT differences) between the defined subtypes. We used Tukey's *post-hoc* test when equal variances were assumed and Tamhane's T2 *post-hoc* test otherwise. To compare the RTs of the three NP subtypes with their corresponding non-NP control conditions, we used independent *t*-tests. The alpha level was set at 0.05 for all statistical tests. The commercial statistical package SPSS 13.0 for Windows (SPSS 13.0; SPSS Inc., Chicago, IL, USA) was used for all statistical analyses.

## Results

### Stroop effect

Sixty-six participants performed the Stroop task using the materials reported by Raz et al. (2002). The ANOVA revealed significantly different RTs between the Stroop types [*F*_(2,15,639)_ = 380.44, *p* < 0.0001]. *Post-hoc* analysis revealed significant Stroop effect (RT_incongruent_ − RT_congruent_; *p* < 0.0001), Stroop interference (RT_incongruent_ − RT_neutral_; *p* < 0.0001), and Stroop facilitation (RT_neutral_ − RT_congruent_; *p* < 0.0001). Mean RTs in the congruent condition (664 ± 205.6 ms) was shorter than those of neutral (702 ± 213.4 ms) and incongruent (790 ± 287.7 ms) conditions.

### Heterogeneity of NP effect

**Figure 2:**
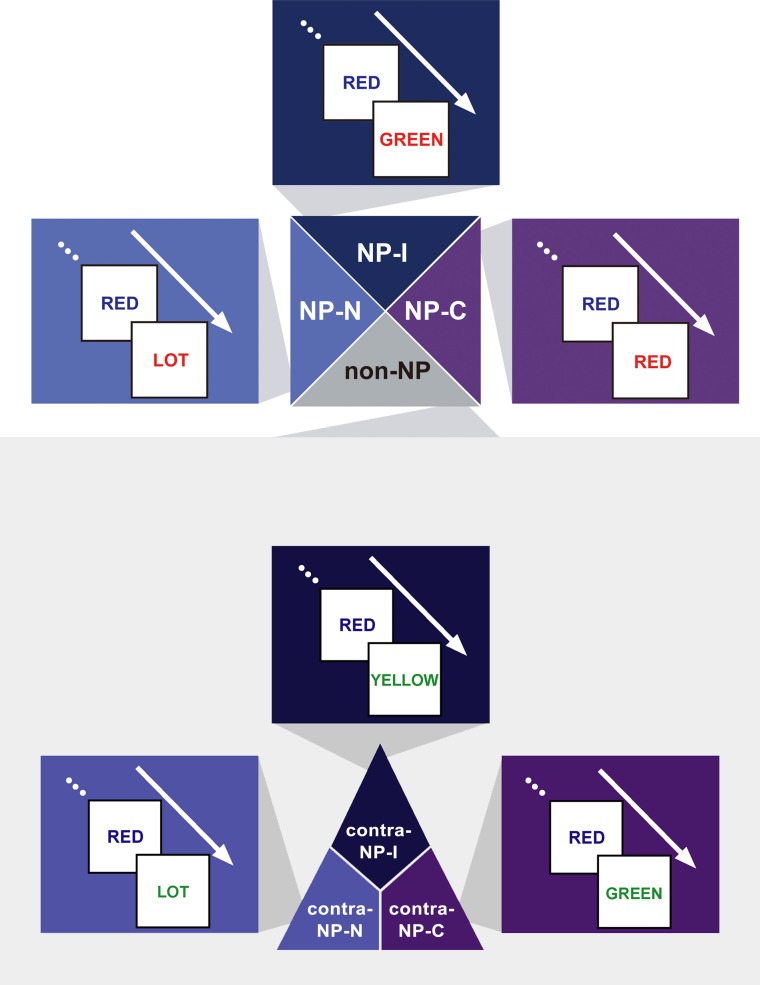
**Exemplars of the three NP subtypes of the NP effect—NP-I, an incongruent prime–incongruent probe stimulus pair, NP-N, an incongruent prime–neutral probe pair, and NP-C, an incongruent prime–congruent probe pair—and their three non-NP control conditions—contra-NP-I, contra-NP-N, and contra-NP-C**. Non-NP control pairs have the same Stroop subtypes with their matching NP subtypes.

We hypothesized that the degree of disinhibition in NP effect depends on the combination of successive stimuli (i.e., prime-probe stimulus pairs) among congruent, neutral, and incongruent items in NP trials. Given that the definition of NP requires an incongruent prime, we divided NP into three subtypes as a function of the probe: NP-I, NP-N, and NP-C. NP-I is a pair having an incongruent probe, which commonly defines the conventional NP type. NP-N and NP-C are prime-probe stimulus pairs having a neutral and congruent probe, respectively. Figure 2 provides a sketch of the three NP subtypes.

Based on this NP subtype definition, we investigated the possible presence of differential disinhibition in NP effect by comparing RTs for the three NP subtypes. We found that NP-I, the most demanding NP subtype, elicited significantly longer RTs than NP-N and NP-C [*F*_(2,1123)_ = 28.02, *p* < 0.0001], which indicates that NP-I has the largest degree of inhibition among NP subtypes. From the *post-hoc* analyses, we observed that NP-I was significantly longer compared with NP-N (*p* < 0.0001) and NP-C (*p* < 0.0001), but RTs for NP-N and NP-C were not significantly different (*p* = 0.436; Table 1). We also compared RTs for the three NP subtypes with their corresponding non-NP control conditions [i.e., a non-distracting prime with incongruent (NP-I), neutral (NP-N), and congruent (NP-C) probes] for the Stroop data. For example, “RED in blue—YELLOW in green” is for the corresponding non-NP control of NP-I, “RED in blue—LOT in green” is for the corresponding non-NP control of NP-N, and “RED in blue—GREEN in green” is for the corresponding non-NP control of NP-C (Figure 2).

**Table 1:** **Comparison of RTs for each NP condition in the human behavioral data**.

		**Mean RTs ± *SD* (ms)**
NP	NP-I	830 ± 274.9
	NP-N	708 ± 210.1
	NP-C	730 ± 234.1
Non-NP	Incongruent (contra-NP-I)	787 ± 288.6
	Neutral (contra-NP-N)	702 ± 213.6
	Congruent (contra-NP-C)	659 ± 202.4

RT, reaction time; NP, negative priming; SD, standard deviation.

**Figure 3:**
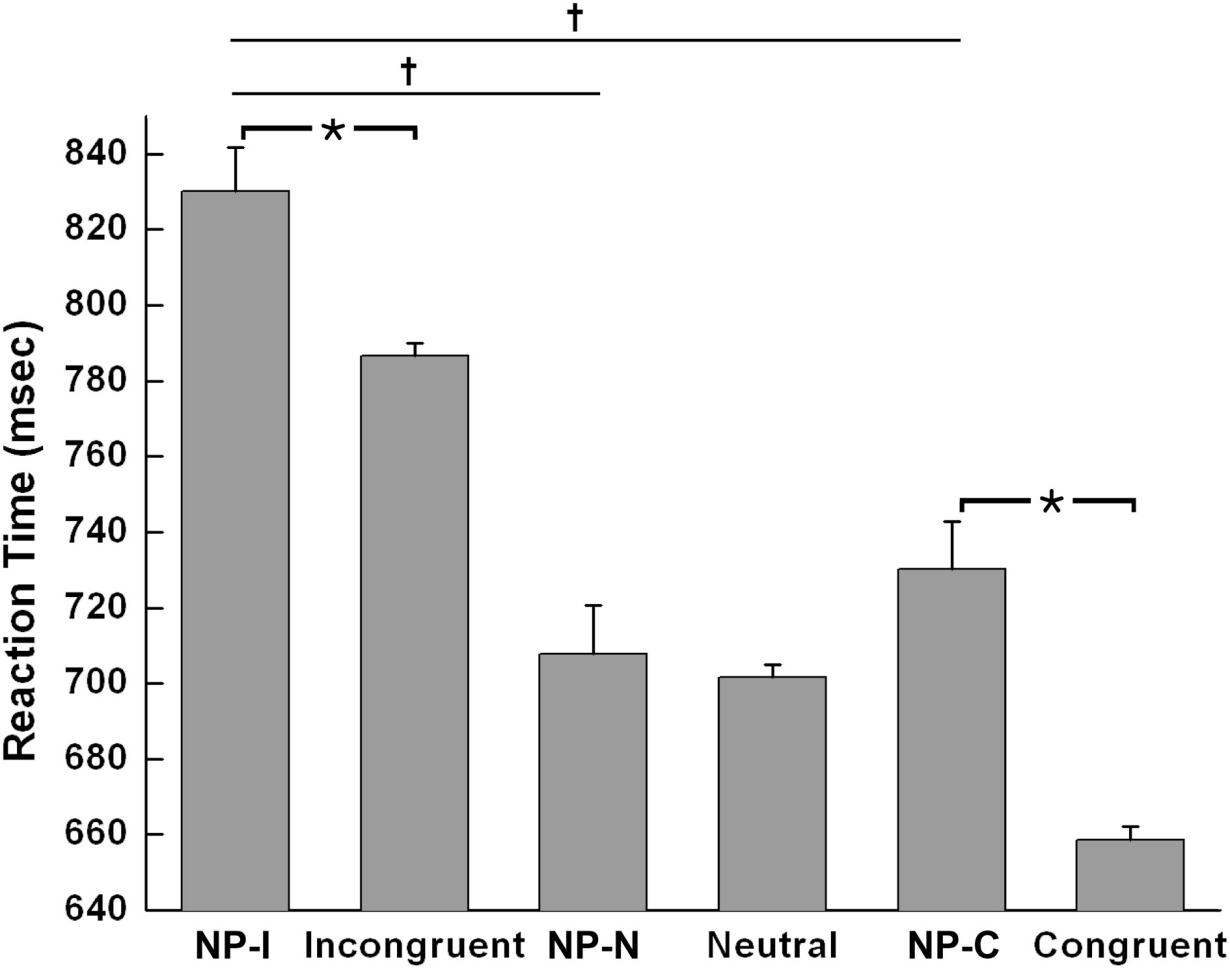
**Mean RTs of human participants during Stroop task performance for each NP subtype and its corresponding non-NP control**. NP-I compared with incongruent and NP-C compared with congruent types showed significant RT differences. Among three NP subtypes, NP-I showed the longest RT, but the RTs of NP-N and NP-C were comparable. Standard errors of each type are represented as error bar; ^*^p < 0.01, ^†^*p* < 0.0001.

The Student *t*-test revealed that NP-I and NP-C required significantly longer RTs than their respective non-NP conditions, as shown in Figure 3 [NP-I: *t*_(5158)_ = 2.96, *p* < 0.005; NP-C: *t*_(5065)_ = 6.40, *p* < 0.0001], while NP-N trials were comparable to the controls [*t*_(5413)_ = 0.54, *p* = 0.59]. Table 1 summarizes these results. These results indicate the presence of distinct NP subtypes due to differential disinhibition of NP effects depending on stimulus combinations of prime-probe pairs.

### Computer simulations of differential NP inhibition

To investigate whether the observed differential disinhibition of NP involves selective attention or/and working memory, we estimated the RTs using conventional PDP and PDP-WM models and compared them with the behavioral data. The number of iterations required to produce a response in the model corresponded to the RT for each trial. Because the PDP-WM model represents both selective attention and working memory, whereas the PDP model represents selective attention, the fit of these models with the behavioral data determines the possible involvement of selective attention and/or working memory in differential disinhibition of NP (see the Methods section for detailed structure and algorithms of the two models).

**Figure 4:**
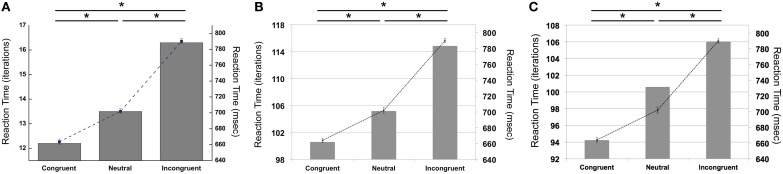
**Mean RTs (left *Y*-axis) of the computational models during Stroop task performance**. **(A)** PDP reproduces the Stroop effect, Stroop interference, and facilitation. **(B)** PDP-WM also reproduces the Stroop effect, Stroop interference, and facilitation. **(C)** Without temporal-storage layer, PDP-refined, a conventional parallel distributed processing model that running average was discarded, can also reproduce Stroop effect, Stroop interference and Stroop facilitation. Human behavioral data (right *Y*-axis) are displayed using dotted lines; ^*^p < 0.0001.

Our simulations revealed that the PDP model exhibited the Stroop effect, Stroop interference, and Stroop facilitation as shown in Table 2 [*F*_(2,49,465)_ = 2613.91, *p* < 0.0001; *post-hoc*: *p* < 0.0001 for all combinations]. Furthermore, comparison of RTs in the PDP model with the behavioral data revealed that the PDP model reproduced Stroop performance well (Figure 4A). Both PDP-WM model and PDP-refined model also yielded all three effects [i.e., the Stroop effect, Stroop interference, and Stroop facilitation; PDP-WM: *F*_(2,99,405)_ = 439.55, *p* < 0.0001, *post-hoc*: *p* < 0.0001 for all combinations; PDP-refined: *F*_(2,98,994)_ = 863.36, *p* < 00.0001, *post-hoc*: *p* < 0.0001 for all combinations; Table 2]. Comparison of RT values from the PDP-WM model with the actual RTs revealed that the PDP-WM model was also a good predictor of the behavioral data (Figures 4B,C). In other words, both with working memory, the PDP-WM model, and without the working memory component, the PDP and PDP-refined models, Stroop performance can be simulated. This result suggests that working memory involvement does not have a critical role in generating the Stroop effect, Stroop interference, or Stroop facilitation.

**Table 2:** **Comparison of RTs for each Stroop condition between conventional PDP and PDP-WM models**.

	**Conventional PDP**	**PDP-WM**	**PDP-refined**
Congruent	12.2 ± 3.7	100.6 ± 32.6	94.2 ± 23.6
Neutral	13.5 ± 4.3	105.2 ± 77.5	100.6 ± 29.1
Incongruent	16.3 ± 6.1	114.9 ± 38.0	106.0 ± 40.0

Reaction time (RT) for network model indicates the number of iteration to produce a response (mean RTs ± SD); PDP, parallel distributed processing model; PDP-WM, parallel distributed processing model with working memory; SD, standard deviation.

**Figure 5:**
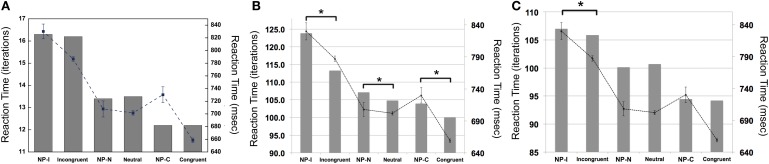
**Mean RTs (left *Y*-axis) of the computational models for the NP effect. (A)** PDP did not reproduce the NP effect in any of NP subtypes compared with each of corresponding non-NP type. **(B)** PDP-WM did reproduce differential disinhibition of NP. All NP subtypes showed significantly delayed RTs compared with their matching non-NP subtypes. **(C)** PDP-refined model cannot simulate significant negative priming effects. Human behavioral data (right *Y*-axis) are displayed using dotted lines; ^*^p < 0.0001.

To examine whether the PDP model could capture differential disinhibition of NP, we compared the RTs between the three NP subtypes and their corresponding non-NP controls in the PDP model and compared them with those found in the behavioral data. Our simulation showed that the PDP model did not produce any significant NP effect [NP-I vs. contra-NP-I: *t*_(29,633)_ = 0.94, *p* = 0.35; NP-N vs. contra-NP-N: *t*_(9830)_ = −0.36, *p* = 0.72; NP-C vs. contra-NP-C: *t*_(9999)_ = −0.11, *p* = 0.91; Table 3]. In addition, the RT values for NP-C differed from the corresponding behavioral data, as shown in Figure 5A. These results suggest that selective attention alone does not account for differential NP inhibition.

**Table 3:** **Comparison of RTs for each NP condition between conventional PDP and PDP-WM models**.

		**Conventional**	**PDP-WM**	**PDP-refined PDP**		
NP	NP-I	16.3 ± 6.0	123.8 ± 138.2	107.0 ± 45.1
	NP-N	13.4 ± 4.3	107.1 ± 31.5	100.1 ± 29.3
	NP-C	12.2 ± 3.7	103.9 ± 34.3	94.5 ± 23.6
Non-NP	Incongruent (contra-NP-I)	16.2 ± 6.1	113.2 ± 60.3	105.9 ± 39.0
	Neutral (contra-NP-N)	13.5 ± 4.3	104.8 ± 39.1	100.7 ± 29.1
	Congruent (contra-NP-C)	12.2 ± 3.7	100.0 ± 32.2	94.2 ± 23.6

Reaction time (RT) for network model simulation indicates the number of iterations necessary to produce a response (mean RT ± SD); NP, negative priming; PDP, parallel distributed processing model; PDP-WM, parallel distributed processing model with working memory.

On the other hand, the PDP-WM model did reveal significant differences in RT values between each NP subtype and its corresponding non-NP control [NP-I vs. contra-NP-I: *t*_(59,760)_ = 12.0, *p* < 0.0001; NP-N vs. contra-NP-N: *t*_(19,990)_ = 3.2, *p* < 0.01; NP-C vs. contra-NP-C: *t*_(19,652)_ = 6.0, *p* < 0.0001], as summarized in Table 3. Furthermore, the RT profiles for NP-I, NP-N, and NP-C were comparable to the behavioral data). NP-I was significantly higher than the other NP subtypes, but as in the human data, NP-N and NP-C showed no difference (NP-I vs. NP-N: *p* < 0.0001; NP-I vs. NP-C: *p* < 0.0001; NP-N vs. NP-C: *p* = 0.49; Figure 5B). These results suggest that, together with selective attention, working memory is responsible for differential NP disinhibition. While the PDP-WM model used temporal storage of short-term memory, the PDP model relied on a running average to keep previous activations (see Table 4 for PP effect simulated from the PDP-WM model as a sanity check).

**Table 4:** **Positive priming effect simulated in the PDP-WM model**.

	**Original RT**	**Positive priming RT**	**Statistics**
Congruent	97.55 ± 28.35	86.91 ± 22.32	*t*_(1005)_ = 14.75, *p* < 0.0001
Neutral	102.54 ± 39.58	97.21 ± 92.29	*t*_(3025)_ = 9.79, *p* < 0.0001
Incongruent	110.00 ± 62.35	89.17 ± 24.72	*t*_(1023)_ = 12.84, *p* < 0.0001

To test the role of working memory component in the simulation, we assessed the PDP-refined model (Figure 5C). The PDP-refined model did not show significant NP effects, except it showed RT difference between NP-I and contra-NP-I [NP-I vs. contra-NP-I: *t*_(59,561)_ = 2.6, *p* < 0.05; NP-N vs. contra-NP-N: *t*_(19,918)_ = −1.0, *p* = 0.32; NP-C vs. contra-NP-C: *t*_(19,512)_ = 0.6, *p* = 0.6]. Thus, we confirmed that the NP effect we observed from the PDP-WM model was generated from the time-delayed layer.

### Working memory weight and differential NP inhibition

**Figure 6:**
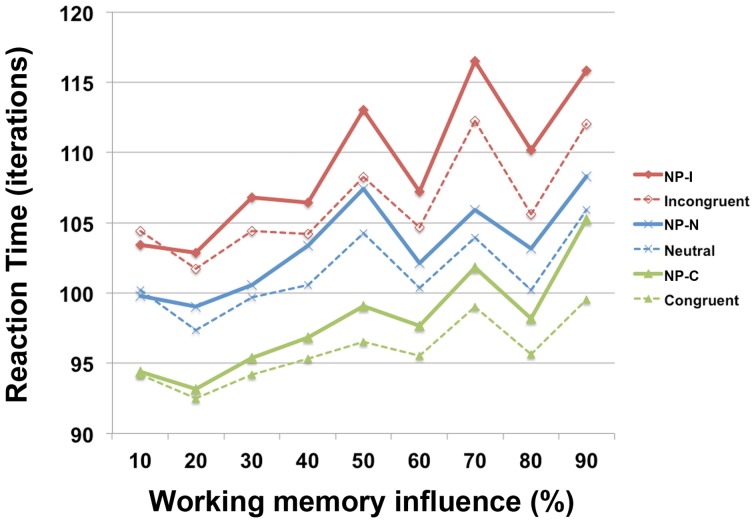
**RT profiles of PDP-WM during Stroop performance as a function of the influence of temporal-storage capacity ranging from 10 to 90%**.

To investigate the involvement of working memory in differential NP inhibition, we modulated the degree of interaction (*w*_delay_) between output units and temporal storage units from 10 to 90% in the PDP-WM model and monitored the degree of NP inhibition (i.e., RT values for each NP subtype). Note that a 0% degree of interaction in the PDP-WM model will turn this network into a conventional PDP model that is deprived of the running average algorithm (PDP-refined). We found that the NP effect and the differences in degrees of NP inhibition became smaller as the influence of working memory on the output layer diminished (Figure 6; Table 2). This result demonstrates the importance of the involvement of working memory in differential disinhibition within NP. In addition, this result is consistent with previous behavioral studies reporting that individuals with decreased working memory capacity show reductions in the NP effect (Conway et al., 1999; Long and Prat, 2002).

## Discussion

The current study tested and showed the possible presence of differential disinhibition in the NP effect that depends on combinations of successive prime-probe stimulus pairs in the Stroop task. We found differential longer RTs in each NP subtype, although they had the same amount of cognitive inhibition (i.e., the same prime). We constructed a PDP model that employed a selective attention mechanism alone and a PDP-WM model that employed both selective attention and working memory and examined the different speeds of disinhibition of NP in these formal models during Stroop task performance. We show that only the PDP-WM model successfully reproduced both the Stroop effect and differential NP disinhibition.

The presence of subtypes of NP depending on prime-probe combinations has been suggested in different contexts (Lowe, 1979; Tipper and Cranston, 1985; Moore, 1994b; Neill et al., 1994; Schrobsdorff et al., 2007; von Hecker and Conway, 2009). Particularly, Schrobsdorff and his colleagues (Schrobsdorff et al., 2007) divided priming trials into the following four subtypes based on targets and distractors in prime-probe pairs: PP cases in which the target is repeated in the prime-probe pair, other positive priming (PP2) in which the distractor is repeated in the prime-probe pair, NP in which the distractor in the prime stimulus becomes the new target in the probe stimulus (NP-I herein), and other NP2 in which the target and the distractor in prime stimulus exchange their roles, becoming the new distractor and the new target, respectively, in the probe stimulus (also categorized as NP-I herein) (c.f., NPSO, a prime followed by a single-object probe whose target was a distractor of the prime, is similar with NP-N in the current study). Schrobsdorff and his colleagues developed the CISAM that assumed a threshold that adapts to the global mean activity level is the single underlying mechanism for both positive and NP. They demonstrated that the CISAM captured the essential features of the positive and NP effects observed in behavioral priming experiments. In addition, various studies have examined the dependence of the NP effect on the nature of the probe stimulus (Lowe, 1979; Tipper, 1985; Moore, 1994a). These studies employed the terms attended-repetition, neutral, and ignored-repetition to report that NP effect could be abolished or reversed according to the existence of the conflict within the probe. The sub-types of NP effects were defined based on the relationship between the prime and the probe stimulus. Thus, the effects of disinhibition level (pure probe stimulus effect) were not disentangled from attention level in explaining differential NP effects. On the other hand, in the current study, we focused on different degrees of disinhibition that are only modulated by the probe to classify the NP subtypes. According to our definition, we observed NP differences based on different Stroop types of probe stimuli following equal levels of inhibition (equal prime stimulus). Parallel to the previous studies, we suggest that differential NP inhibition and disinhibition effects should be considered in any comprehensive theoretical account of NP phenomenon.

Our findings suggest that memory retrieval is implicated in differential NP disinhibition, which is consistent with previous NP studies [for review, (Fox, 1995; MacLeod and MacDonald, 2000)]. Despite the early predominance of the selective disinhibition theory (Tipper, 1985; Fuentes and Tudela, 1992; Engle et al., 1995; Malley and Strayer, 1995; Strayer and Grison, 1999), recent studies propose that, rather than construing NP as arising from ignoring a previous stimulus (Wood and Milliken, 1998; MacDonald et al., 1999), it may result from processing mismatches across successive presentations of the repeated item (Park and Kanwisher, 1994; Chiappe and MacLeod, 1995; MacDonald and Joordens, 2000), in line with memory-based accounts (MacLeod and MacDonald, 2000). Behavioral and neuroimaging studies also support the notion that individuals endowed with greater working memory capabilities have increased NP effects relative to those with less efficient working memory systems (Conway et al., 1999; Le Van Quyen et al., 2001; Long and Prat, 2002; Egner and Hirsch, 2005; Rothermund et al., 2005). The episodic memory retrieval models (Neill and Valdes, 1992; Frings et al., 2007) and their variants (Park and Kanwisher, 1994; Milliken et al., 1998) suggest that the possible mechanisms underlying differential NP inhibitions presumably hinge on the trial onset triggering retrieval of the prime from episodic memory along with a “do not respond” tag for the distractor prime. This tag conflicts with the current processing to respond to the former distractor and thus requires removal, which results in the time delay observed in NP conditions. Furthermore, similarities between the prime and probe mainly trigger the retrieval of the reaction to the prime; thus, the more similar the trials are, the stronger the retrieved representation is (Frings et al., 2007; Schrobsdorff et al., 2007). However, our model supports the notion that actively maintained information about the prime itself (the most immediate history via working memory), rather than retrieval of a tag for the specific stimulus, induces differential disinhibition in the response to the probe. To confirm the involvement of working memory retrieval in differential NP inhibition effects and consequent NP subtypes, neuroimaging investigations of working memory systems during Stroop task performance are required in the future (see Figure 6).

It is worth noting the conceptual similarities of our model and one of the recently suggested model from Schrobsdorff et al. (2012a), the GMNP. First, both models implement both distractor-inhibition and memory retrieval algorithms to account for NP effect. Selective attention affects the activation level differentially for a target and a distractor. Memory retrieval intervened at the stage before action selection. Second, in both models, decisions are made when the activation levels between the target and distractor are dissociable based on a decision threshold. Even though mathematical description was different, adaptive threshold algorithm has similar concept with evidence accumulator in the current study on singling out the winning signal. However, the differences between the models are still not negligible. First, GMNP uses episodic memory retrieval, which compares similarity signal from the present stimuli and the past sequence and changes the route (by blocking or facilitating the weights) of the process. PDP-WM uses working memory, which occurs automatically and affects decision process of the present stimulus (by exciting or inhibiting the nodes). Second, GMNP focused on suggesting a generalized model that covers various dimensions of tasks. However, because of the generalization approach, the model had a large number of free parameters. PDP-WM focuses on suggesting a plausible mechanism (working memory) of NP within the classical Stroop paradigm, explained by parallel-distributed processing model. Despite of different degree of freedom and mathematical descriptions, the two models are not opposed to one another in supporting the roles of distractor-inhibition and memory on NP effect.

The PFC is a key candidate for the neural substrates subserving differential NP inhibition during Stroop task performance. Known to control the planning of complex behaviors, executive functions, selective attention, and short-term memory (O'Reilly et al., 1999; Miller and Cohen, 2001; Cohen et al., 2002), the PFC actively processes necessary information and inhibits less relevant information in the face of excess inherently distracting information and is responsible for cognitive control (Cohen and O'Reilly, 1996; Constantinidis et al., 2002; Konishi et al., 2005; Mayr et al., 2006). The PFC is involved in the inhibitions of prolonged set interference (Konishi et al., 2005), inhibitions in guiding or inhibiting responses (Constantinidis et al., 2002), and RT costs from demands of switching between task rules for efficiency (Mayr et al., 2006). Functional neuroimaging studies have demonstrated that several regions of the PFC are selectively activated relative to the neutral condition (Steel et al., 2001; Egner and Hirsch, 2005; Wright et al., 2005, 2006), and similar results have been found for the bilateral lingual gyri (Vuilleumier et al., 2005), the anterolateral temporal cortex (Steel et al., 2001; de Zubicaray et al., 2006), and the inferior parietal lobule (Steel et al., 2001). In line with these findings, our PDP-WM model that included a time-delayed layer, the compartment that executes working memory as the PFC, exhibited complex NP effects, supporting the idea that the PFC is a neural substrate for causing different degrees of disinhibition in NP effects during the Stroop task.

The current study has several limitations. First, RT differences between NP subtypes in the current study were described as global characteristics rather than at the level of individual subjects. We assumed that there are general features of NP effects that are independent of trial repetition and that the participants have comparable capacities of cognitive functions (e.g., working memory). However, we did not directly examine their general cognitive skills. Thus, the absolute magnitudes of the NP effects in each subtype should be interpreted with careful consideration. Second, we heuristically set the working memory weight to 80% in the PDP-WM model. Even though the current model did not implement neuronal spike model, the working memory weight can be considered as decay of preserved activation (memory) between the trials (i.e., ISI). According to the simulated results about the influence of working memory, RT differences between each combination of NP subtype and its corresponding non-NP subtype gradually increased as a function of working memory influence, which is consistent with Neill and Valdes (1992). Non-monotonic changes in RTs might be due to (normally distributed) randomness in the evidence accumulator. However, further research employing a quantitative approach is needed to optimize the parameters that will lead to further understanding of human cognition. Third, because the artificial neural network was defined to simulate cognitive processes in an abstract level (not simulating neuronal firing), it is hard to make one-to-one match between the model parameters (e.g., trained weights of the model) and the biological system (brain connectivity). The PDP-WM is based on a hypothesis that working memory would have a key role generating NP. Thus, we have to take a priori degree of freedom of the model into account when interpreting the results.

Nevertheless, the current study suggests a formal computational model that integrates two previously modeled functions of the PFC—attention and working memory—into a single framework to account for the NP phenomena. By introducing the time-delayed layer, we showed that working memory and its retrieval affect NP effects through the most immediate inhibition but not through the long term history of events. Our collective findings suggest that, together with selective attention, working memory is responsible for transmitting the inhibited or excited information about the prime stimulus to the processing of the next trial (probe stimulus) and the elicitation of differential NP disinhibition.

### Conflict of interest statement

The authors declare that the research was conducted in the absence of any commercial or financial relationships that could be construed as a potential conflict of interest.
